# Utilising active play interventions to promote physical activity and improve fundamental movement skills in children: a systematic review and meta-analysis

**DOI:** 10.1186/s12889-018-5687-z

**Published:** 2018-06-26

**Authors:** Avril Johnstone, Adrienne R. Hughes, Anne Martin, John J. Reilly

**Affiliations:** 1 Physical Activity for Health Group, University of Strathclyde, Glasgow, Scotland; 2 MRC/CSO Social and Public Health Sciences Unit, University of Glasgow, Glasgow, Scotland

**Keywords:** Active play, Fundamental movement skills, Cognition, Weight status, Children, Physical activity, Moderate-to-vigorous physical activity, Systematic review

## Abstract

**Background:**

Children’s physical activity levels are low and efforts to improve their physical activity levels have proven difficult. Freely chosen and unstructured physical activity (active play) has the potential to be promoted in a variety of settings and potentially every day of the year in contrast to other physical activity domains, but active play interventions are an under-researched area. Therefore, the primary aim of this systematic review was to determine the effect of active play interventions on children’s physical activity levels, particularly moderate-to-vigorous intensity physical activity (MVPA), and fundamental movement skills (FMS).

**Methods:**

Studies were included if they were solely or  predominantly active play randomised, or cluster randomised controlled trials that targeted children aged 3–12 years. They had to report on at least one of the following outcomes: objectively measured physical activity, FMS, cognition and weight status. During December 2016, four databases (PE Index, SPORTDiscus, Medline and ERIC) were searched for relevant titles. Duplicates and irrelevant titles and abstracts were removed. The included studies had their quality assessed using the Effective Public Health Practice Project (EPHPP) tool. Suitable studies were combined in a meta-analysis using a random-effect model. A narrative synthesis was conducted for all outcomes.

**Results:**

Of the 4033 records, 91 studies were eligible for full text screening, of which 87 were removed, leaving four studies (representing five papers). The meta-analysis of two studies highlighted there was no significant effect of active play interventions on MVPA. However, the narrative synthesis suggested that active play interventions may increase total volume of physical activity. Only two studies examined the effect of active play interventions on children’s FMS, one study examined effects on weight status and none examined effects on cognition.

**Conclusions:**

Due to the small number of eligible studies and their heterogeneity, the review could not draw firm conclusions on the effect of active play interventions on children’s physical activity levels. High-quality active play interventions, targeting different times of the day (school and after school) in different populations and settings, and with a wider range of outcomes, are required to determine the potential of active play.

## Background

Engaging in regular physical activity from a young age offers wide ranging health benefits including, reduced risk of cancer, overweight and obesity, depression and diabetes [1, 2]. However, many children from the most high-income countries are not achieving the recommended minimum of 60 min of moderate-to-vigorous physical activity (MVPA) per day [3–5]. Efforts to encourage children to engage in physical activity have tended to focus on four domains: active transportation, recess, physical education (PE) and sports. Recent systematic reviews have suggested that the amount of MVPA being accumulated in these domains is limited, particularly as these domains are largely school-based, and schooldays represent little more than half of all days in a year [6–10]. Community and home-based interventions to promote physical activity are less common, despite the potential for interventions outside of school [11]. One novel area of research is the role active play may have on the contribution to children’s habitual physical activity and MVPA levels [12–15].

Active play is ‘a form of gross motor or total body movement in which young children exert energy in a freely chosen, fun, and unstructured manner’ [16]. Active play could potentially be influenced by multiple levels from policy to environment (school/ pre-school and safe places to play outdoors), to those who influence active play (teachers, parents and peers) to the children themselves, which provides potential opportunities to target active play interventions. Furthermore, it is often engaged in outdoors, which is associated with higher habitual physical activity and MVPA levels, as shown in recent observational studies and systematic reviews [13, 17, 18]. Active play might have the potential for greater population wide gains on habitual physical activity and MVPA levels compared to other domains of physical activity [15]. It is a relatively unrestrictive domain of physical activity: it can be engaged in before school, during school, after school, when schools are on holiday, and often requires no specific infrastructure [12–15].

In low-middle-income countries where physical activity levels are often higher than in high-income countries, children tend to spend more time in active play [5]. However, in high-income countries, it is not clear how often children engage in active play as public health surveillance of this domain is poor. For example, only 17/38 participating countries were able to assign a grade to active play in the recent Active Healthy Kids Global Matrix [4, 5]. It is thought that with the emergence of screen time activities and parental safety concerns, many children are not engaging in active play every day. Therefore, interventions may be required to promote active play in childhood [19, 20].

Active play may generate additional benefits beyond increasing MVPA and physical activity levels, including improved fundamental movement skills (FMS), weight status and cognitive performance [15, 21–24]. FMS are the basic skills children should be competent in such as jumping, running, catching and throwing, and are related to children’s physical activity levels, for example, if children have good FMS they are more likely to be physically active [25–28]. Active play may be a promising way of developing FMS in children [21, 22]. Furthermore, Pesce et al. suggested that active play may improve children’s cognitive performance, particularly if it is combined with activities that develop FMS (or motor skills) [23].

Despite the potential for active play to increase children’s physical activity levels and improve their FMS, cognitive performance and weight status, we are unaware of any systematic review of interventions to promote active play in children [15, 21, 23]. Therefore, the primary aim of this systematic review was to determine the effect of active play interventions in increasing children’s physical activity levels and improving FMS, and to characterise the interventions used. The secondary aim was to determine the effect of active play interventions on improving cognitive performance and weight status in children.

## Methods

### Literature search and inclusion

The present systematic review is reported following the PRISMA statement for conducting systematic reviews and meta-analyses. The protocol was registered on PROSPERO on the 20th January 2017 (http://www.crd.york.ac.uk/PROSPERO/display_record.php?ID=CRD42017055530).

Four relevant electronic databases, MEDLINE, SPORTDiscus, PE Index and ERIC were searched during December 2016. The search strategy followed the PICOS (population, intervention, comparison, outcomes and study design) framework. The inclusion and exclusion criteria are detailed below. The search was limited from 2000 to 2016 given that active play is an emerging area of research, and the search was for studies that used objective measurement of physical activity (which only became available in the late 1990’s). The authors also restricted the search to English language studies only due to the impracticalities of translating papers. An example of a search strategy for the MEDLINE database is provided in Table 1, which was adapted for the three other databases. Full literature search details are available from the corresponding author on request.

**Table 1:** Search Strategy in Medline

child*.tw.	
(boy* or girl*).tw.	
youth*.tw.	
(pupil* or student* or schoolchild* or primar*).tw.	
(young adj2 (person* or people)).tw.	
Elementary*.tw.	
Kindergarten*.tw.	
Grade*.tw.	
1 or 2 or 3 or 4 or 5 or 6 or 7 or 8 or 9	
“Play and Playthings”/	
“active play*”.tw.	
(outdoor adj2 play*).tw.	
“outdoor play*”.tw.	
“physically active play*”.tw.	
physical* activ* play*.tw.	
(outdoor adj2 activ* adj2 play*).tw.	
(unstruct* adj2 activ*).tw.	
“playground”.tw.	
“recess”.tw.	
(recreation* adj1 activ*).tw.	
(activ* adj2 free adj2 play).tw.	
“active free play*”.tw.	
“physical play”.tw.	
playground*.tw.	
Parks, Recreational/	
11 or 12 or 13 or 14 or 15 or 16 or 17 or 18 or 19 or 20 or 21 or 22 or 23 or 24 or 25 or 26	
Randomized Controlled Trial/	
Control Groups/	
compar*.tw.	
Control*.tw.	
(control* adj1 trial*).tw.	
“random* cont*”.tw.	
allocat*.tw.	
28 or 29 or 30 or 31 or 32 or 33 or 34	
Exercise/	
(physical* adj2 activ*).tw.	
exercis*.tw.	
(physic* adj2 fitness).tw.	
(physic* adj2 endurance).tw.	
(physical activity adj2 (level* or intensit* or energy expenditure)).tw.	
“MVPA”.tw.	
moderate-to-vigorous.tw.	
“moderate to vigorous”.tw.	
fitness.tw.	
physical* activ*.tw.	
(cardio adj2 respiratory adj2 fitness).tw.	
Motor Activity/	
“fundamental movement skill*”.tw.	
movement skills.tw.	
(motor adj2 skills).tw.	
(gross adj2 motor adj2 development).tw.	
(motor adj2 development).tw.	
“gross motor skill*”.tw.	
(Motor adj2 compet*).tw.	
(Motor adj2 develop*).tw.	
(motor adj2 proficiency).tw.	
Locomotor.tw.	
object control.tw.	
(movement adj2 compet*).tw.	
Cognition/	
learning.tw.	
“executive function*”.tw.	
(cognitive adj2 performance).tw.	
“inhibition”.tw.	
(working adj2 memory).tw.	
“memory”.tw.	
(self adj2 regulation).tw.	
“self-regulation”.tw.	
behav*.tw.	
“attainment”.tw.	
“Weights and Measures”/	
Body Weight/	
(body adj2 mass adj2 index).tw.	
“body mass index”.tw.	
BMI.tw.	
(weight adj2 status).tw.	
(overweight or obesity).tw.	
Adiposity.tw.	
Fat.tw.	
36 or 37 or 38 or 39 or 40 or 41 or 42 or 43 or 44 or 45 or 46 or 47 or 48 or 49 or 50 or 51 or 52 or 53 or 54 or 55 or 56 or 57 or 58 or 59 or 60 or 61 or 62 or 63 or 64 or 65 or 66 or 67 or 68 or 69 or 70 or 71 or 72 or 73 or 74 or 75 or 76 or 77 or 78 or 79 or 80	
10 and 27 and 35 and 81	
limit 82 to yr. = “2000 -Current”	
limit 83 to English language	

References were imported into endnote and duplicates were removed at which point one researcher screened the titles and abstracts with another researcher checking 10% of the included and excluded articles. Two researchers then independently screened relevant full text articles. If the researchers could not agree during any part of the screening process, then a third researcher was consulted to resolve the disagreement. Reference and citation lists of the final included papers were examined to find any potential eligible studies missed during the database search.

#### Population

Apparently healthy children and adolescents aged- 3-12 years old were included in the present systematic review as this is the age that children tend to engage in active play [29]. Studies of children with any intellectual, physical or cognitive disabilities, which may impair their ability to engage in active play, were excluded.

#### Intervention

For inclusion in the present review, the intervention had to consist of either solely active play (as defined above) or if the intervention was multi-component, active play had to be the predominant component [16]. Active play was determined to be the predominant component if the time allocation for active play was reported as being greater than or equal to any of the other intervention components. Decisions on whether to include or exclude papers were based on the description of the intervention in the paper. The intervention could take place in a range of settings including school (including pre-school), community (located in a community centre, park or streets) or home-based interventions [30]. Any intervention that was related solely or largely to sport, physical education or active video games was excluded because these activities do not fall into the definition of active play.

The intervention must have lasted at least 8 weeks in duration, to minimise the impact of short-term/ novelty effects. Two school-based systematic reviews looking at the effect of physical activity interventions only included studies that lasted at least 12-weeks in duration [31, 32]; therefore, due to active play being an emerging area of research, the authors lowered the duration to 8-weeks to try to capture as many active play interventions as possible.

#### Comparison

The intervention had to be compared to a comparison or control group, who received either no treatment, another physical activity intervention, other lifestyle intervention, wait list control or attentional control. Uncontrolled studies were excluded*.*

#### Outcome

There were two primary outcomes, physical activity and FMS. Studies looking at the effect on physical activity must have measured habitual or total physical activity, or MVPA using an objective method (for example using an accelerometer) to be included. Recess interventions, which have been subject to many systematic reviews previously, were excluded if they only measured changes in physical activity during the recess period. The present study aimed to review evidence on physical activity over a greater period of time, enough to represent school day, habitual or total physical activity, or MVPA. Physical activity measured using observation or questionnaire, or an objective measurement that does not give an intensity (for example pedometers), or studies that measured a small period of the day such as recess interventions were excluded.

Fundamental movement skills had to be measured using a valid and reliable assessment (for example the Test of Gross Motor Development-2 or Movement Assessment Battery for Children-2). Studies that self-reported FMS were excluded.

Secondary outcomes of the present systematic review were cognitive performance and weight status. Cognitive performance should have been measured using direct observation (e.g. time on task), questionnaires or laptop-based assessments of standard cognitive tasks (such as a flanker test) [33]. Weight status had to be measured objectively using a stadiometer and electronic scales or any other valid assessment of height and weight. Studies that reported self-report weight status were excluded.

#### Study design

Studies included in the systematic review had to be randomised controlled trials, cluster randomised controlled trials or comparison studies where the sample had been randomised. Non-randomised controlled trials were excluded.

### Data extraction

Data were extracted by one of the authors and checked by a second. All authors agreed on what data should be extracted, which included study information (e.g. study design), population details, intervention characteristics (e.g. setting, duration and frequency of the intervention), details of the comparison or control group, outcomes (e.g., how and when outcomes were measured) and results.

In instances where data were missing, or additional information was required for the eligible studies, the study authors were contacted to provide the relevant information.

Two authors were contacted to determine if the study interventions and designs met the inclusion criteria; one for additional information on the intervention and the other to ascertain whether the study was randomised [34, 35]. Another author was contacted for additional data, which they were unable to provide [36].

### Data analysis and synthesis

A meta-analysis was conducted using the Review Manager 5.3 software for the MVPA outcomes only using random-effect models. Due to the small number of studies and the heterogeneity of the data, the authors could not conduct a meta-analysis for the other outcomes. Combined effect sizes were weighted by the sample size and standard error of the primary study. Effect sizes were reported as mean differences and 95% confidence intervals. The statistical heterogeneity was assessed using the I^2^ statistic.

A narrative synthesis was conducted on outcomes in which a meta-analysis could not be conducted (total physical activity, FMS, cognition and weight status); with interventions described by reviewing the type, duration and setting. The authors of the present systematic review considered doing sub-group analysis by the type of active play (indoor or outdoor), age (pre-school or school) and setting (school, community or home); however, due to the small number of eligible papers, this was not possible.

### Quality assessment

Two reviewers independently assessed the quality of the eligible papers using the quality assessment tool of the Effective Public Health Practice Project (EPHPP) [37]. In instances where the two reviewers could not agree on the quality of the paper, a third reviewer was consulted. Briefly, the EPHPP tool assesses selection bias, study design, consideration of confounders, blinding, data collection method, withdrawals and dropouts using a scoring system that rates the quality of each of these components as strong, moderate or weak [37]. The EPHPP tool has strong inter-rater reliability and construct validity [37, 38].

## Results

### Characteristics of eligible studies

**Fig. 1:**
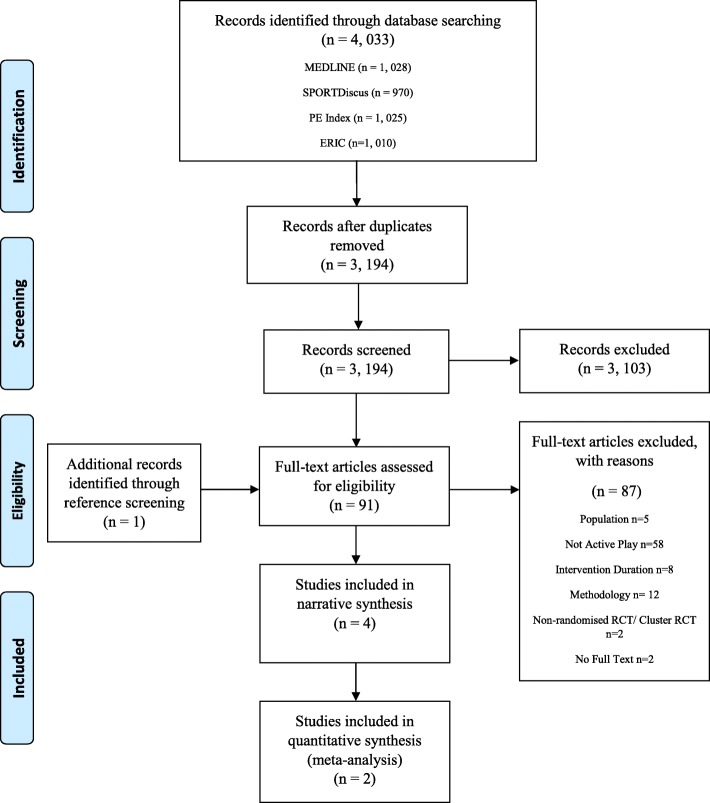
Flow diagram of number of articles retrieved during the literature search and study selection

The PRISMA flow diagram is presented in Fig. 1 [39]. Of the 4033 articles identified from four databases, 91 were eligible for full text screening. Of these, four studies (representing five papers) were eligible for inclusion. One further paper was identified when searching references of the four included studies [24], however this paper was a part of a study already included, but reported different outcomes [22]. Reasons for exclusion are reported in Fig. 1.

An overview of the included studies is presented in Table 2. One study was conducted in Canada [22, 24], one in Australia [34] and the other two in Europe (Italy and England) [35, 36]. The eligible studies had a relatively small number of participants ranging from 76 to 221 in total (intervention and control group) [22, 24, 34–36]. Adamo et al. and Goldfield et al. [22, 24] (mean age 3.4 years), and O’Dwyer et al. (mean age 3.8 years) targeted pre-school children [36], whereas Engelen et al. [34] (mean age 6.0 years) and Tortella et al. (intervention- mean 5.6 years; comparison – 5.7 years) targeted school-aged children [35]. One study was conducted in a pre-school setting [22, 24], two were school-based [34, 35] and the final study was based in a community setting [36]. All of the included studies were cluster randomised controlled trials [22, 24, 34–36]. The duration of the intervention varied from 10-weeks, [35, 36], 13-weeks [34] and 6-months [22, 24].

**Table 2:** Overview of included studies

Author, year and setting	Study design	sample size intervention/ control	Age (range or mean ± SD), sex (n or % m/f	Intervention duration	Outcome(s)	Intervention Details	Comparison / Treatment	EPHPP Quality Rating
Adamo et al. 2016 [22]& Goldfield et al. 2016 Canada [24]	Cluster RCT	40/ 43	Intervention: age (3.4 ± 0.3), sex (18/22) Control: age (3.4 ± 0.4), sex (23/20)	6-months	PA measured using an Actical accelerometer FMS measured using the TGMD-2 Weight status measured using stadiometer and digital scales.	Two 3-h training workshops to childcare providers. Workshops encouraged childcare providers to provide more outdoor active play (manuals provided). Basic equipment provided. Bi-weekly booster sessions provided.	Regular childhood curriculum	Moderate
Engelen et al. 2013 Australia [34]	Cluster RCT	113/ 108	Intervention: age (6.0 ± 0.6), sex (59/54) Control: age, (6.0 ± 0.6), sex (60/48)	13-weeks	PA measured using an ActiGraph accelerometer	Loose parts equipment provided in the playground. Two-hour information session for staff on playground duties aimed at highlighting the benefits of active free play.	Standard break times	Weak
O’Dwyer et al. 2012 England [36]	Cluster RCT	33/ 43	All: age (3.8 ± 0.6), sex (52%/48%),	10-weeks	PA measured using an ActiGraph accelerometer	5 sessions over 10 weeks. 60 min delivery. The first 20 min children and parents were separated. Parents received educational component and children participated in active play. Final 40 min both children and parents participated in active play together.	No treatment	Weak
Tortella et al. 2016 Italy [35]	Cluster RCT	71/ 39	Intervention: age (5.6 ± 0.31), sex (41/30) Control: age (5.7 ± 0.3), sex (22/17)	10-weeks	FMS measured using Movement ABC	30 min of free play and 30 min of structured activities once a week for 10 weeks in the playground	No treatment	Weak

*RCT* randomised controlled trial, *SD* standard deviation, *n* number

Two studies assessed objectively measured physical activity as the only outcome [34, 36], one assessed FMS only [35] and the final study (representing two papers) objectively measured physical activity, FMS and weight status [22, 24]. None of the four studies included assessed the child’s cognitive performance.

### Intervention descriptions

A range of active play interventions was utilised in the four included studies. The interventions by Adamo et al. and Goldfield et al. took place in child-care centres and involved two-three hour workshops for the care providers to encourage active and outdoor play for the children (3–5 years) [22, 24]. During these workshops, the care providers received an active play manual and equipment, which aimed to facilitate active and outdoor play with pre-school aged children [22, 24].

Engelen et al. and Tortella et al. utilised playground settings for their active play interventions [34, 35]. Engelen et al. provided loose play equipment (e.g. tyres, crates, recycled plastic and fabric) for children in a school playground [34]. Tortella et al. brought children from the local kindergarten to the playground for the intervention once a week for one hour [35]. Tortella et al. then divided the playground into motor skill specific areas (balance, dexterity, mobility) where the children played for 10 min each and the remaining 30 min was free play [35].

Finally, O’Dwyer et al. delivered a parent and child (pre-school children-mean age 3.8 years) active play intervention in the community [36]. Over the 10-week period, the families had five contact sessions lasting one-hour delivered by play workers: for the first 20 min, the parents and children were separated and the parents received an educational component and the children participated in active play, and for the final 40 min, they participated in active play together [36].

### Quality assessment

Table 3 presents the quality rating of each of the four studies graded by the EPHPP tool. Three [34–36] of the four included studies were rated as ‘weak’ using the EPHPP tool and the other as ‘moderate’ [22, 24]. Studies were typically rated weak for the ‘selection bias’, ‘study design’ and blinding categories.

**Table 3:** Quality Assessment

Study	Selection Bias	Study Design	Confounders	Blinding	Data Collection Methods	Withdrawals and Drop-outs	Total
Adamo et al. 2016 [22]& Goldfield et al. 2016 [24]	Moderate	Strong	Weak	Moderate	Strong	Strong	Moderate
Engelen et al. 2013 [34]	Weak	Weak	Moderate	Moderate	Strong	Strong	Weak
O’Dwyer et al. 2012 [36]	Weak	Weak	Weak	Weak	Strong	Strong	Weak
Tortella et al. 2016 [35]	Weak	Weak	Weak	Weak	Strong	Weak	Weak

### Effects of the interventions

#### Moderate-to-vigorous intensity physical activity

**Fig. 2:**
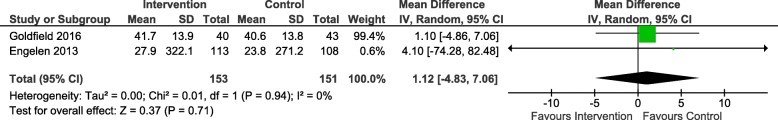
Effect of active play interventions on minutes/pre-school or school day spent in moderate-to-vigorous physical activity (MVPA)

Figure 2 presents the results from the meta-analysis on MVPA from two studies [24, 34]. These two studies were found to be homogenous (I^2^ = 0%) but there was no significant effect on MVPA (*p* = 0.71; MD = 1.12, 95%CI: -4.83, 7.06) when the two studies were pooled.

#### Total volume of physical activity

Three of the included studies examined the effects of the active play interventions on total physical activity, but we could not conduct a meta-analysis because the data the authors reported varied (minutes/school day physical activity, counts per minute, weekday and weekend minutes/day) [24, 34, 36].

Goldfield et al. [24] found that the intervention group increased their pre-school day total physical activity by 19 min (95% CI: 9, 30) whereas the control group decreased their pre-school day total physical activity by 3 min (95% CI: -12, 6) [24].

O’Dwyer et al. [36] reported a significant increase in total physical activity during the weekday and weekend in the intervention group compared to the control group [36]. The intervention increased total physical activity by 5 min (95% CI: 3, 9) during the weekday and by 10 min (95% CI: 2, 18) during the weekend compared to the control group [36].

Engelen et al. did not find a significant increase in school day accelerometer counts per minute in the intervention group compared to the control (95% CI: -144, 116) [34]. At baseline, total counts per minute in the intervention group were 216 (SE = 5) and this was 217 (SE = 5) at follow up, whereas at baseline the control groups total counts per minute were 199 (SE = 5) and this was 197 at follow up (SE = 5) [34].

#### Fundamental movement skills

Two of the studies included in the present systematic review analysed the effects of an active play intervention on children’s FMS [22, 35]. A meta-analysis could not be conducted due to the different methods used to measure FMS.

Adamo et al. utilised the Test of Gross Motor Development-2 to assess children’s fundamental movement skills [22]. The authors found that both the gross motor quotient score (95% CI: 0.7, 10.7; *p* = 0.025;) and percentile (95% CI: 2.2, 24.5; *p* = 0.020) significantly increased in the intervention group compared to the control [22]. The intervention group had a mean change of 4.2 (95% CI: 0.5, 7.9) for gross motor quotient score and a mean change of 9.6 (95% CI: 1.3, 18.0) for percentile [22]; the control group had a mean change of − 1.5 (95% CI: -4.8, 1.8) for gross motor quotient score and a mean change of − 3.7 (95% CI: -11.1, 3.7) for percentile [22].

Tortella et al. used a combination of assessments to measure FMS, including the Test of Motor Competence, Movement Assessment Battery for Children and the Test of Physical Fitness [35]. They assessed performance in a range of skills including one leg balance, balance on beam, balance on platform, heel to toe walking task and throwing a medicine ball [35]. They found significant improvements in all of the aforementioned skills in the intervention group compared to the control group apart from one-leg balance (right foot) [35].

#### Cognitive performance

None of the studies included in the present systematic review investigated the effects of active play interventions on children’s cognitive performance.

#### Weight status

One of the studies included in the present systematic review investigated the effects of an active play intervention on children’s weight status [24]. This study found that participating in a 6-month active play intervention did not significantly decrease BMI-z score in the intervention group compared to the comparison group [24], although the intervention group did have a decrease in BMI-z score of − 0.2 (95% CI: 0.0, 0.4; *p* = 0.087) [24].

## Discussion

The present systematic review highlighted that there is limited randomised controlled research on active play interventions, despite the potential benefits they may have on children’s physical activity levels, FMS, cognition and weight status: only four eligible studies (described in 5 separate papers) were identified [22, 24, 34–36]. A meta-analysis was conducted on the MVPA outcome only and showed there was no significant effect [24, 34]. However, the evidence base on utilising active play as a domain in childhood physical activity interventions seems very small at present.

Research efforts aimed at increasing levels of physical activity and MVPA in children have largely focussed on other school-based domains, including recess, PE and active commuting. Comparable systematic reviews looking at other domains of physical activity have included a greater number of eligible RCTs and cluster RCTs, for example; recess interventions (*n* = 9), elementary school PE (*n* = 13) and active commuting (*n* = 32) [8–10]. Janssen recently suggested that active play has the biggest potential for increasing children’s physical activity levels due to its unrestrictive nature [15], i.e. active play can be promoted before, during and after school and when schools are on holiday.

Three of the included studies utilised a pre-school or school setting and only one utilised a community setting. Schools provide a good opportunity to promote physical activity as they have access to all children, in particular, children who might not otherwise engage in physical activity [40, 41]. However, given that children only spend half of their days in school, other settings (community or home) outside school hours need further attention [40, 41].

Active play is often engaged in outdoors, and outdoor time is associated with higher MVPA levels [13, 17]. A recent observational study in English children suggested that engaging in physical activity after school hours is important to increasing children’s habitual physical activity and, in particular, MVPA levels [18]. Specifically, children who engaged in physical activity (most likely through active play) after school 3–4 times per week achieved a mean of 8-min more MVPA per day [18].

The meta-analysis conducted in the present systematic review suggested that active play interventions have little effect on increasing MVPA levels. However, this meta-analysis was conducted on only two studies, these were rated as weak and moderate quality, and utilised different types of accelerometers and accelerometer cut points to determine time spent in MVPA. Furthermore, the interventions differed in duration and type with one study lasting 13-weeks and focussing on recess in primary schools, and the other lasting 6-months and aimed at promoting outdoor active play in a pre-school setting [24, 34].

It might be that active play has a greater effect on children’s total volume of physical activity in addition to, or instead of, any effects on MVPA. Although a meta-analysis could not be conducted on the studies which measured total volume of physical activity as an outcome, three studies included in the present systematic review found improvements in total physical activity in the active play intervention groups. Both O’Dwyer et al. and Goldfield et al. found a statistically significant increase in total physical activity (minutes/day) in the intervention group compared to the control [24, 36]. O’Dwyer et al. conducted a 10-week community-based intervention and utilised an ActiGraph accelerometer to measure total physical activity [36]. The intervention group increased their total physical activity by 5 min and 10 min during the weekday and weekend, respectively [36]. Goldfield et al. conducted a longer pre-school intervention lasting 6-months and found that pre-school day total physical activity increased by 19 min in the intervention group [24]. Despite these two studies varying in intervention design, setting and duration, and the device used to measure physical activity (ActiGraph and Actical), they do suggest that promoting active play may be a potentially useful way to increase the total volume of habitual physical activity.

A recently published non-randomised controlled study, by the authors of the present systematic review, also found improvements in percent time in light physical activity and MVPA. This was a school-based intervention in which classes received two active play sessions per week for 5-months, which elicited a 16 and 3% increase in light physical activity and MVPA, respectively [21]. However, these findings need to be confirmed by a fully powered future definitive cluster RCT [21].

In addition to potential effects of active play on physical activity, increased engagement in active play also has the potential to improve FMS, and low FMS among children in the developed world is a topic of increasing interest [25–28]. In the present systematic review, two included studies examined the effect of an active play intervention on children’s FMS (or gross motor development) [22, 35]. These two studies utilised different intervention designs with one opting for a pre-school setting and encouraging more outdoor active play opportunities and the other offering kindergarten children a one-hour per week active play session at a local park [22, 35]. These two active play interventions utilised different methods of assessing children’s FMS but both significantly improved FMS in the intervention group compared to the control [22, 35]. However, these two studies were of weak to moderate quality, which highlights the need for more high-quality studies to test the extent to which active play interventions can improve FMS.

The development of FMS might improve both physical activity and weight status: Stodden presented a conceptual model proposing that developing FMS in children increased their physical activity levels and thus in turn promoted healthy weight in children [28, 42]. Janssen also recommended that interventions aimed at reducing levels of overweight and obesity in children should include an active play component, as the potential gain in terms of energy balance seemed greater than for interventions that targeted other domains of physical activity in children [15, 20].

Only one study in the present systematic review looked at the effect of an active play intervention on reducing overweight and obesity [24]. Although, this study did not find a significant intervention effect the intervention group did have an apparent decrease in BMI-z score [24]. Future active play intervention studies could consider including measures of weight status as outcomes, and preferably, body composition rather than simple proxies for body composition as these are more likely to be able to detect intervention effects [43].

None of the included papers in the present review looked at the effects of active play interventions on cognitive performance. Research has suggested a likely association between physical activity levels, in particular, MVPA and improved cognition [33, 44, 45]. Furthermore, it has been suggested that active play might be particularly beneficial to improving children’s cognition as it is likely to be engaged in at a high intensity (MVPA), often takes place outdoors and involves cognitively engaging activities [23, 46]. The combination of MVPA and cognitive engagement may be particularly helpful for the development of cognitive skills relevant for school performance [23, 44]. Future studies should consider assessing cognitive and/or educational outcome measures, and additionally, measures of other social and emotional outcomes as these might also be improved by engaging in more active play. Active play is an enjoyable experience for children, which is important because it relates to their likelihood of being physically active throughout their life course and thus acquiring good FMS and improving their cognition [23].

As the evidence base for active play is limited, there is huge potential for future research into its effects on physical activity and other outcomes. Studies aimed at exploring the barriers and facilitators to active play have highlighted that many parents are concerned about children’s safety and therefore limit their active play opportunities, and this is particularly prevalent in more deprived communities [19]. Given that parents who engage in higher levels of physical activity are also likely to have children who are more physically active, then future intervention research should consider a parental component, as they are the decision makers in most children’s lives. Only one study in the present systematic review had a parental component [36].

During the systematic review process, the authors were aware that the vague nature of the definition of active play (provided above) could be problematic. A recent systematic review by Truelove et al. aimed to provide a working definition for active play [16]. Key elements of this definition are ‘freely chosen’ and ‘unstructured’; however, all of the included studies in the present systematic review involved adult involvement in varying amounts, ranging from providing more opportunities for active play (increased outdoor time, more equipment to encourage free play) within a (pre) school context to playworkers facilitating an intervention. Due to poor surveillance of active play (discussed above), we cannot be certain the amount of time children spend engaging in active play, but given low levels of physical activity among children in developed countries, it appears to be low [5]. Therefore, if active play has huge potential for increasing physical activity, there may need to be some adult involvement to provide more opportunities for active play but we need to consider whether this really does conform to the definition of active play. Time allocation of active play within an intervention, i.e. does most of the intervention consist of ‘active play’ may support this or it might be best to consider further sub-definitions of active play, such as ‘active free play’, ‘facilitated active play’ etc.

Since the present systematic review found only a small amount of published randomised controlled evidence, a search for ongoing trials was carried out using appropriate terms in www.clinicaltrials.gov and www.isrctn.com in November 2017. Three ongoing, potentially eligible randomised controlled studies were identified; two will assess physical activity levels, FMS, weight, and cognition and the other will assess physical activity levels only. These ongoing studies will see the evidence base for active play increase modestly in the near future.

### Review and evidence base strengths and weaknesses

The present systematic review aimed to consider the most robust intervention studies by only including randomised controlled study designs, which has limited the number of included studies by excluding other study designs. For practical reasons, we were unable to review studies in languages other than English, which may have limited the number of included studies. While all eligible studies were RCTs, three of the four included studies were rated weak and one was rated moderate using the EPHPP tool, so the published evidence base is small and not of the highest quality. The present systematic review was also limited to searching from 2000 to the end of 2016. The rationale for this is that we included only studies with objectively measured physical activity outcomes (only available since the late 1990s), active play is an emerging area of research, and the most recent evidence was considered to be the most generalisable. We also did not review the evidence surrounding social and emotional effects of active play, which needs further exploration in interventional research as well as a future systematic review.

Overall the present systematic review suggests that few RCTs have tested the efficacy of active play interventions in children, have only tested efficacy over a relatively short period, and have only examined efficacy for a very limited number of outcomes. Furthermore, it seems that none of the included studies in the present systematic review assessed the fidelity of their respective interventions, meaning that we could not determine why these interventions did not have the desired effect on MVPA and FMS. Future RCTs should also assess the fidelity of the interventions to determine if they were implemented as intended. This would provide essential information to the field by providing a deeper understanding as to why interventions might not provide the desired result.

## Conclusions

The present systematic review aimed to determine the effect of active play interventions on children’s physical activity levels, FMS, cognition and weight status. Due to the small number of eligible studies and their heterogeneity, the review could not draw firm conclusions on the effect of active play interventions on these outcomes. High-quality active play interventions, targeting different times of the day (school and after school) in different populations and settings, and with a wider range of outcomes, are required to determine the potential of active play in increasing physical activity levels and improving FMS, cognition and weight status in children.

## Abbreviations

BMI: Body mass index
EPHPP: Effective Public Health Practice Project
FMS: Fundamental Movement Skills
MVPA: Moderate-to-vigorous physical activity
PE: Physical education
PRISMA: Preferred Reporting Items for Systematic Reviews and Meta-Analyses
RCT: Randomised controlled trial
